# No effect of a single supratherapeutic dose of lersivirine, a next-generation NNRTI, on QTc interval in healthy subjects

**DOI:** 10.1186/1758-2652-13-S4-P225

**Published:** 2010-11-08

**Authors:** M Vourvahis, R Wong, NM Ndongo, M O'Gorman, M Tawadrous

**Affiliations:** 1 Pfizer Global Research and Development, New London, USA; 2 Pfizer Research Clinic, Brussels, Belgium; 3 Pfizer Global Research and Development, 50 Pequot Avenue, New London, USA

## Purpose of the study

Lersivirine (LRV) is a next-generation NNRTI currently under investigation in two Phase IIb studies at doses of 500 - 1000 mg QD. *In vitro* analyses suggest that LRV is a weak I_kr_ and CA^2+^ channel blocker. However, dose-ranging Phase I studies have not shown prolongation of QTc interval to date. This study was performed to investigate the effect of a supratherapeutic dose of LRV on QTc interval in healthy subjects, relative to matched placebo and active control.

## Methods

A randomized, single-dose, placebo- and active-controlled 3-way crossover study was performed with 48 healthy adults. Subjects were randomized to receive either LRV (2400 mg), moxifloxacin (400 mg) or placebo for each treatment period with minimum washout of 7 days. Triplicate 12-lead electrocardiogram (ECG) measurements were performed and PK samples collected predose and at 1, 2, 3, 4, 5, 6, 9, 12, and 24 hrs postdose. Vital signs were measured predose, 3 hrs postdose and at discharge. Adverse event monitoring and safety laboratory testing were performed throughout.

## Summary of results

All 48 subjects enrolled were white males (mean age 39.1 yrs, body mass index 25.6 kg/m^2^) and completed the study. Following LRV administration, the upper bound of 90% CI for time-matched adjusted mean differences to placebo in QTcF at each of the time points postdose was below the regulatory threshold of 10 msec, thus satisfying the criteria for a negative thorough QT/QTc study. The highest upper bound of 90% CI occurred at 6 hrs for LRV (3.32, 90% CI 1.5, 5.1). The study was deemed adequately sensitive as the lower bound of the 90% CI for the adjusted mean differences between moxifloxacin and placebo at moxifloxacin's historical T_max_ of 3 hrs was >5 msec (15.29 msec, 90% CI: 13.5, 17.1 msec). No subjects receiving LRV or placebo had a QTcF ≥450 msec, nor did any experience a QTcF increase ≥30 msec from baseline at any time point. In subjects receiving LRV, no clinically significant changes in QRS complex, PR interval, heart rate or blood pressure were observed. PK analysis of blood samples indicated geometric mean (CV%) AUC_last_ 17750 ng.hr/mL (28%) and C_max_ 1727 ng/mL (33%), and median T_max_ 3 hrs. Adverse events were generally mild, with some moderate gastrointestinal events.

## Conclusions

LRV administered as a single 2400 mg supratherapeutic dose did not prolong the QTc interval and no clinically relevant ECG or vital sign changes were observed in healthy subjects.

